# A right pulmonary vein abnormality treated with 3D CT assistance in thoracoscopic surgery for esophageal cancer: a case report

**DOI:** 10.1186/s40792-022-01396-8

**Published:** 2022-03-16

**Authors:** Naoki Kuwayama, Isamu Hoshino, Hisashi Gunji, Toru Tonooka, Hiroaki Soda, Takeshi Kurosaki, Nobuhiro Takiguchi, Yoshihiro Nabeya, Wataru Takayama

**Affiliations:** Division of Gastroenterological Surgery, Chiba Cancer Center, 666-2 Nitona-cho, Chuo-ku, Chiba, 260-8717 Japan

**Keywords:** Esophageal cancer, Thoracoscopic esophagectomy, Thoracoscopy, Anomalous pulmonary vein

## Abstract

**Background:**

Anomalous bifurcation of the right superior pulmonary vein is an important anomaly that should be recognized not only in respiratory and cardiac surgeries, but also in esophageal surgery for the safe performance of surgery. We report a case in which thoracoscopic esophagectomy was safely performed using preoperative three-dimensional computed tomography (3D CT) imaging.

**Case presentation:**

An 81-year-old male patient received an upper gastrointestinal endoscopy, which revealed a 20-cm incisor at the entrance, 43-cm EGJ, and 30-mm large type 1 + IIc lesion between the 23-cm and 26-cm incisors; biopsy showed squamous cell carcinoma (SCC). Contrast-enhanced CT showed wall thickening in the anterior wall of the upper thoracic esophagus, without evidence of multi-organ invasion or lymph node metastasis. In addition, a break in the right pulmonary vein passing dorsal to the right main bronchus and flowing directly into the left atrium was observed, and 3D CT was performed preoperatively to confirm the 3D positioning. Positron emission tomography (PET)–CT showed a high degree of accumulation (SUVmax 19.95) in the upper thoracic esophagus. The patient was diagnosed with upper thoracic esophageal cancer, cT2N0M0 cStage II, and underwent thoracoscopic subtotal esophagectomy (three-region dissection) and gastric tube reconstruction. The dorsal inflow of the pulmonary vein in the right main bronchus, which was recognized on preoperative CT, was confirmed and preserved. The pathological diagnosis was basaloid squamous cell carcinoma, pT1b(SM1)N0(0/58)M0 pStage I. The postoperative course was uneventful, and the patient was discharged on postoperative day 20.

**Conclusions:**

The anomalous bifurcation of the pulmonary vein in the right upper lobe area required attention because of its potential to cause massive bleeding and difficulty in securing the operative field if misidentified and damaged during surgery. Although it is not frequently encountered, it is the bifurcation anomaly that esophageal surgeons must bear in mind due to its severe consequences. Preoperative image-reading and intraoperative manipulation of this vessel are imperative for surgical safety.

## Background

Anomalous bifurcation of the pulmonary veins in the right upper lobe area is relatively uncommon. It is often discussed in respiratory and cardiology surgeries [1, 2]; however, it is also a very important bifurcation abnormality for esophageal surgeons who perform lymph node dissection in the mediastinum. If this vessel is damaged during surgery without being recognized, it may cause massive bleeding and difficulty in securing the field of view; therefore, it is important to recognize it on preoperative imaging. We report a case in which the vessel was recognized by preoperative contrast-enhanced computed tomography (CT) and imaged using three-dimensional (3D) CT, which enabled safe surgery.

## Case presentation

**Fig. 1:**
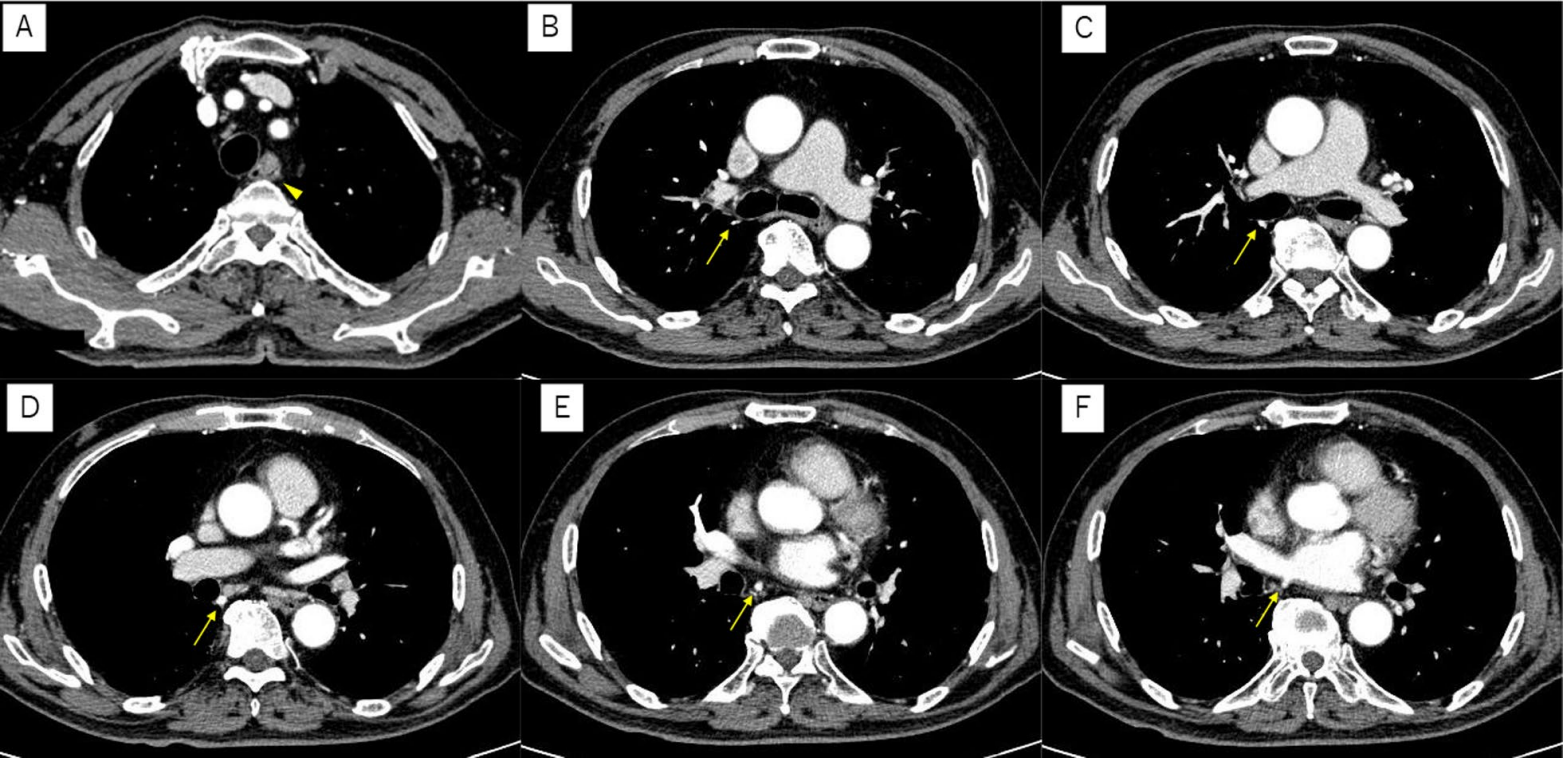
Contrast-enhanced CT findings. **A** Contrast-enhanced CT showed mural thickening of the anterior wall of the upper thoracic esophagus, with no evidence of multi-organ invasion or lymph node metastasis. **B**–**F** A break in the right pulmonary vein passing dorsal to the right main bronchus and flowing directly into the left atrium was observed (arrow in the figure)

**Fig. 2:**
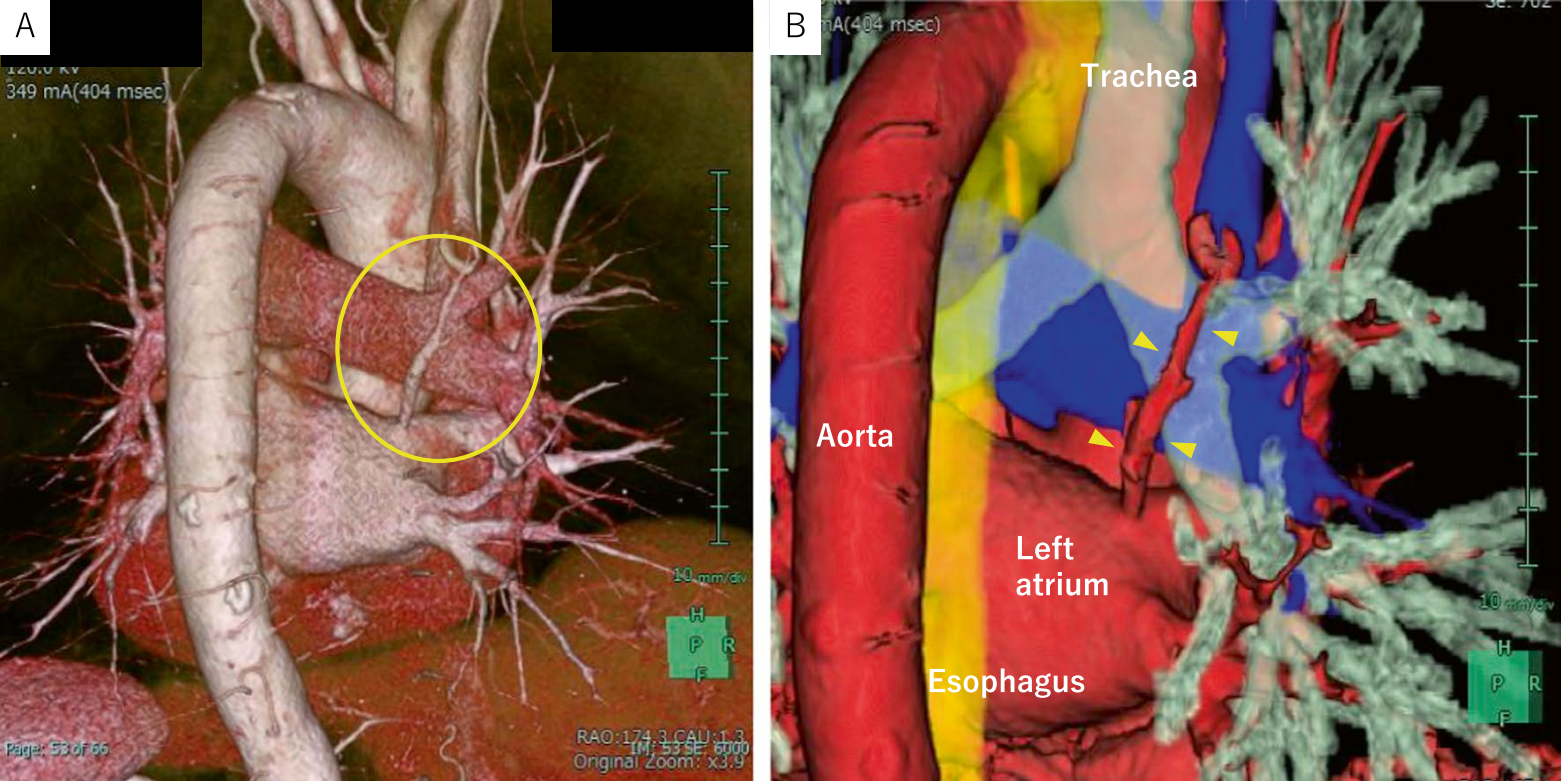
3D CT imaging. **A**, **B** 3D CT was performed preoperatively to confirm the 3D positioning. Arrowhead: The right pulmonary vein passing dorsal to the right main bronchus and flowing directly into the left atrium

**Fig. 3:**
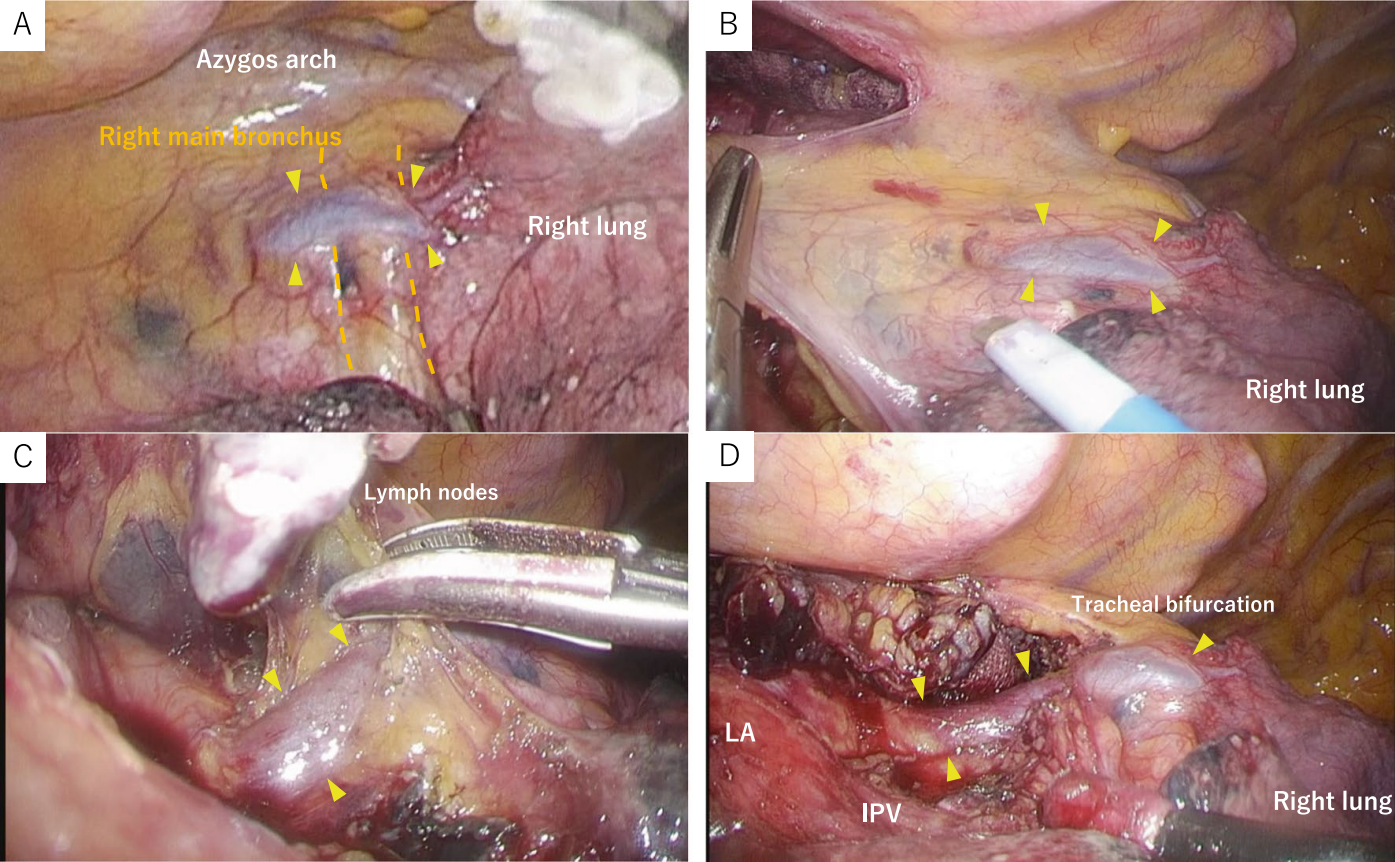
Operative findings. **A**–**D** The lymph nodes at the tracheobronchial bifurcation (#107, #109R, #109 L) were contiguously dissected from the epicardial surface, and the right main bronchus (dotted line) dorsal inflow pulmonary vein (arrowhead) was dissected and preserved. *LA* left atrium, *IPV* inferior pulmonary vein

An 81-year-old man, during a follow-up of asbestos lung disease at his previous doctor's office, showed an elevated lesion in the esophagus using a CT scan. Upper gastrointestinal endoscopy revealed type 1 advanced esophageal cancer in the mid-thoracic esophagus, and the patient was referred to our department. Upper gastrointestinal endoscopy revealed a 20-cm incisor at the entrance, a 43-cm EGJ, and a 30-mm type 1 + IIc lesion between the 23-cm and 26-cm incisors, and a biopsy revealed squamous cell carcinoma (SCC). Contrast-enhanced CT showed mural thickening of the anterior wall of the upper thoracic esophagus, with no evidence of multi-organ invasion or lymph node metastasis (Fig. 1A). In addition, a break in the right pulmonary vein passing dorsal to the right main bronchus and flowing directly into the left atrium was observed (Fig. 1B–F), and preoperative 3D CT was performed for better three-dimensional positioning during lymph node dissection (Fig. 2). Positron emission tomography (PET)–CT showed a high degree of accumulation (SUVmax 19.95) in the upper thoracic esophagus. The patient was diagnosed with upper thoracic esophageal cancer, cT2N0M0 cStage II. The standard treatment is preoperative chemotherapy plus surgery, but we decided to treat him with surgery alone, without preoperative chemotherapy considering his age and other factors. In response to the above diagnosis, thoracoscopic subtotal esophagectomy (three-region dissection) and gastric tube reconstruction were performed. The dorsal inflow of the pulmonary vein of the right main bronchus, which was recognized on preoperative CT, was confirmed. The lymph nodes at the tracheobronchial bifurcation (#107, #109R, #109 L) were contiguously dissected from the epicardial surface, and the right main bronchus dorsal inflow pulmonary vein was dissected and preserved (Fig. 3). The pathological diagnosis was basaloid SCC, pT1b(SM1)N0(0/58)M0 pStage I. The postoperative course was uneventful, and the patient was discharged on postoperative day 20.

## Discussion

The recognition of pulmonary vein anomalies is important for the safe performance of esophageal surgery. Various abnormalities have been reported to exist in the pulmonary veins [1, 2]. Anomalous pulmonary veins passing through the right dorsal bronchus were reported by a radiologist in 1984 [3], and have been discussed mainly in the field of respiratory and cardiology surgeries, where a catheter ablation of pulmonary veins is performed for the treatment of atrial fibrillation.

Fetal pulmonary venous plexus develops around the pulmonary primordium, sprouting from the anterior part of the protointestine and initially connected to the protointestinal venous plexus and the body venous system. At about 28 days of fetal life, the common pulmonary vein sprouts posteriorly from the posterior wall of the left atrium and joins the pulmonary venous plexus. As the pulmonary veins begin to traffic with the left atrium, traffic with the proximal plexus or body vein disappears. If the connection between the pulmonary veins and the left atrium is blocked before this, the pulmonary venous blood has no place to return and it is forced to return to the right atrium via the nearby body veins, and the primordial venous plexus (superior vena cava and coronary sinus), resulting in an abnormal pulmonary venous return [4].

Recently, it has been shown that myocardial progenitor cells in the cardiac inflow tract and cells in the pulmonary vascular plexus are derived from common progenitor cells (cardiopulmonary mesoderm precursors), suggesting that they are related to the pathogenesis of abnormal pulmonary venous return [5]. There have been several reports of abnormal reflux of the pulmonary veins in the right upper lobe. Asai et al. reported 22 cases (3.0%) of anomalous pulmonary veins running dorsal to the middle bronchial trunk and flowing into the superior pulmonary vein by reviewing the chest CT of 725 cases [4]. Similarly, Kim et al. also reported 10 (3.6%) out of 280 cases of rupture flowing into the right superior pulmonary vein [1]. Shiina et al. reported an anomalous pulmonary venous return in the right upper lobe area and summarized the variations in the reflux sites [6].

**Fig. 4:**
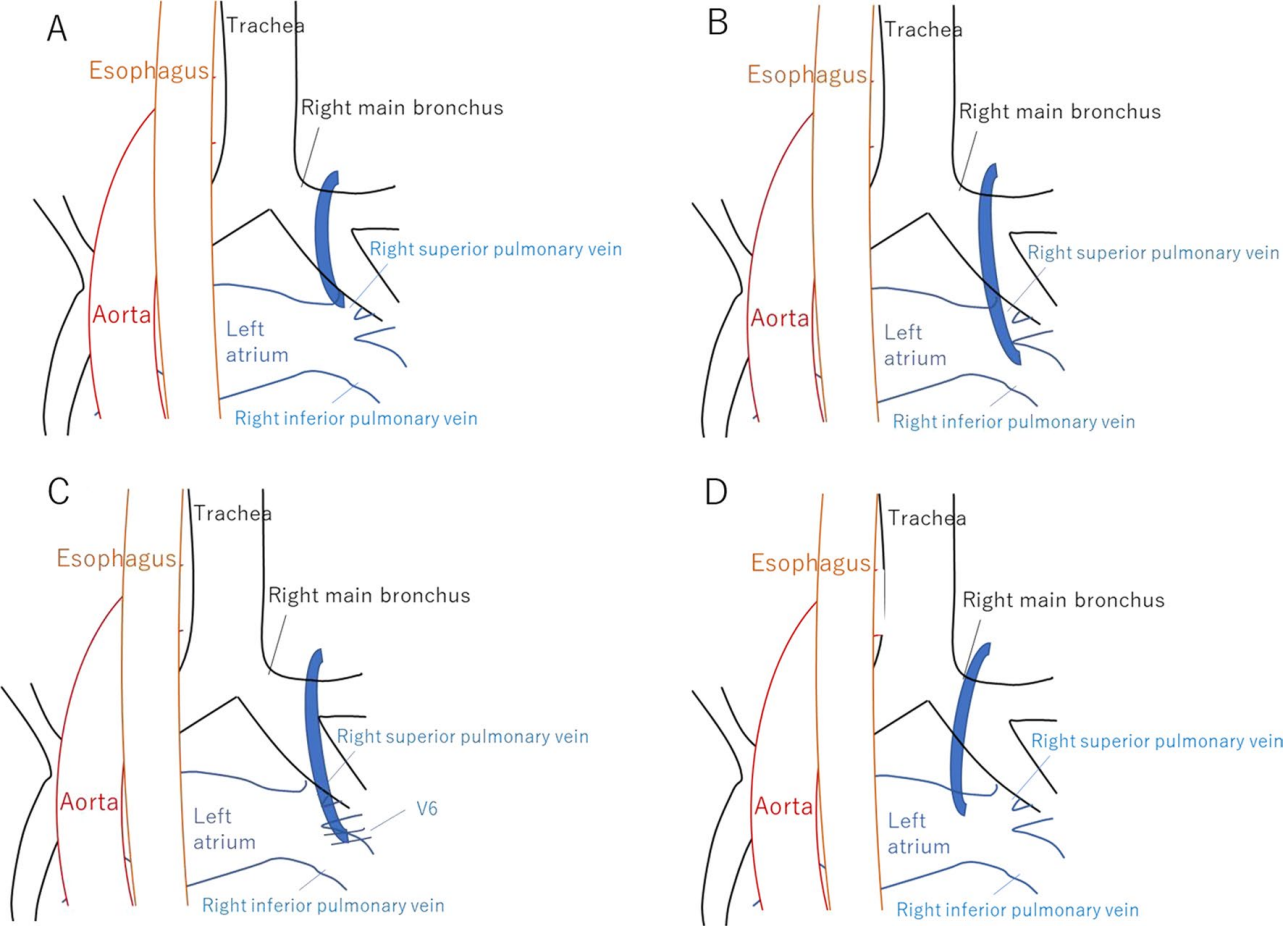
The variation of the inflow site of the pulmonary vein. **A** The cases of the superior pulmonary vein flowing into the right superior pulmonary vein. **B** The cases of the inferior pulmonary vein. **C** The cases in which the right inferior pulmonary vein formed an independent branch of V6. **D** The cases of direct flow into the left atrium

Variations in the reflux sites include (1) cases of the superior pulmonary vein flowing into the right superior pulmonary vein; (2) cases of the inferior pulmonary vein; (3) a small number of cases in which the right inferior pulmonary vein formed an independent branch of V6 and flowed into it (V6 cases), and (4) cases of direct flow into the left atrium [6] (Fig. 4).

Another important point in the classification of abnormal right upper lobe area pulmonary vein reflux is the part of the dorsal bronchus that it passes through. Akiba et al. reported that 8 out of 10 cases with abnormal right pulmonary vein reflux passed through the dorsal right middle bronchial tube, one passed through the right main bronchus, and one through both [7]. Matsubara et al. reported that 2 out of 700 (0.28%) cases had anomalous right upper lobe area pulmonary vein that runs in the esophageal surgical field [8]. One of the reasons for the lack of reports of anomalous pulmonary venous return in esophageal surgery is that when the rupture is not recognized preoperatively, it does not appear in the operative field of esophageal cancer surgery and cannot be recognized when it passes dorsally on the peripheral side of the right middle bronchial trunk.

There were only four reports of abnormal pulmonary venous return in the right upper lobe region in esophageal cancer, including this case, as far as we could find in PubMed (Table 1) [8–10]. In all cases, lymph node dissection was performed while preserving the abnormal pulmonary veins. Matsubara et al. reported that the variant right superior pulmonary vein could be identified, preserved, and dissected during open thoracic surgery for thoracic esophageal cancer. Shiozaki et al. performed a longitudinal esophagectomy with preservation of the pulmonary vein flowing into the left atrium. Onodera et al. reported that preoperative 3D CT and preoperative imaging allowed safe dissection with preservation of the pulmonary veins flowing into the superior pulmonary vein.

**Table 1:** Previous reports of abnormal pulmonary vein in esophageal cancer

Case	Author	Age	Sex	Origin	Inflow	Preoperative diagnosis	Surgical approach
1	Matsubara et al.	57	Male	RUL	Unknown	–	Thoracotomy
2	Shiozaki et al.	74	Male	RUL	LA	–	Transhiatal
3	Onodera et al.	61	Male	RUL	SPV	–	Thoracoscopy
4	Our case	81	Male	RUL	LA	CT	Thoracoscopy

*RUL* right upper lobe, *LA* left atrium, *SPA* superior pulmonary vein

Injury to the pulmonary veins can cause unexpected massive bleeding; especially, in endoscopic surgery, including robotic surgery, because hemostasis becomes difficult to achieve, and the field of vision becomes difficult to maintain. In addition, inadequate drainage of the affected pulmonary vein may cause hemoptysis due to partial pulmonary congestion; therefore, it is necessary to preserve the pulmonary vein [6]. In our case, the right upper lobe pulmonary vein passed through the dorsal side of the right middle bronchial bifurcation and flowed directly into the bifurcation of the left atrial pulmonary vein. The most complicated part of this pulmonary vein variant is the tracheal bifurcation lymph node (107, 109) dissection. These nodes are located dorsal to the pericardium and medial to the main bronchi. Usually, we dissect the lymph nodes from the pericardial surface and then dissect the mediastinal pleura, which is continuous with the trachea. This results in the lymph node being attached only to the medial side of the right main bronchus, which is dissected using an energy device. In cases of a variant right superior pulmonary vein, as in this case, attention should be paid to intraoperative damage to the vein. It is also important to examine this vein for any damage caused by the traction of the right lung.

Preoperative CT and preoperative imaging with 3D CT allowed us to perform a safe surgery. The 3D CT angioplasty is very useful for surgery because it allows us to understand the passage of blood vessel branches that cannot be detected with a regular 5-mm slice CT horizontal section. Although small vessels can be detected by a thin slice of the whole chest, the amount of data obtained is vast. Three-dimensional CT angiography saves storage capacity and is helpful in cost performance.

## Conclusions

Three-dimensional CT angioplasty is very useful for surgery because it provides a 3D view of blood vessel branches. The usefulness of 3D CT has been reported in respiratory surgery, and its active use in esophageal cancer surgery is also desired [11]. The anomalous bifurcation of the pulmonary veins in the right upper lobe is a relatively rare anomaly; however, it is associated with the risk of bleeding due to the possibility of injury during mediastinal dissection in esophageal cancer surgery. Despite not being frequently encountered, preoperative image-reading and the intraoperative manipulation of this vessel will lead to surgical safety in esophageal surgery.

## Data Availability

Data sharing is not applicable to this article, as no datasets were generated or analyzed during the study.

## Abbreviations

SCC: Squamous cell carcinoma
3D: Three-dimensional
CT: Computed tomography
PET: Positron emission tomography
